# Utilizing community InfoSpots for health education: perspectives and experiences in Migoli and Izazi, Tanzania

**DOI:** 10.1093/heapro/daab187

**Published:** 2021-12-13

**Authors:** Christine Holst, Naomi Tschirhart, Bernard Ngowi, Josef Noll, Andrea Sylvia Winkler

**Affiliations:** 1 Centre for Global Health, Department of Community Medicine and Global Health, Institute of Health and Society, University of Oslo, P.O box 1130, Blindern, 0318 Oslo, Norway; 2 Interdisciplinary School of Health Sciences, Faculty of Health Sciences, University of Ottawa, Thompson Hall - THN 136, 25 University Private, Ottawa, ON K1N 7K4, Canada; 3 Oslo Group on Global Health Policy, Department of Community Medicine and Global Health, Institute of Health and Society, University of Oslo, P.O. box 1130, Blindern, 0318 Oslo, Norway; 4 Department of Family Medicine, Chiang Mai University, 239 Huay Kaew Road, Muang District, 50200 Chiang Mai Thailand, Thailand; 5 Muhimbili Medical Research Centre, National Institute for Medical Research (NIMR), 3 Barack Obama Drive, P.O. box 9653, 11101 Dar es Salaam, Tanzania; 6 University of Dar es Salaam, Mbeya College of Health and Allied Sciences, P.O. box 608, Mbeya, Tanzania; 7 Basic Internet Foundation, Gunnar Randers vei 19, 2007 Kjeller, Norway; 8 Department of Technology Systems, University of Oslo, P.O box 70, 2027 Kjeller, Norway; 9 Center for Global Health, Department of Neurology, Technical University of Munich, Ismaninger Straße 22, 81675 München, Germany

**Keywords:** global health, disease prevention, community health promotion, Africa, ICT

## Abstract

Limited access to health education can be a barrier for reaching the Sustainable Development Goals, especially in rural communities in sub-Saharan Africa. We addressed this gap by installing community information spots (InfoSpots) with access to the internet and a locally stored digital health education platform (the platform) in Migoli and Izazi, Tanzania. The objective of this case study was to explore the perspectives and experiences of InfoSpot users and non-users in these communities. We conducted 35 semi-structured interviews with participants living, working or studying in Migoli or Izazi in February 2020 and subsequently analysed the data using content analysis.

The 25 InfoSpot users reported variations in use patterns. Users with more education utilized the platform for their own health education and that of others, in addition to internet surfing. High school students also used the platform for practicing English, in addition to health education. Most InfoSpot users found the platform easy to use; however, those with less education received guidance from other users. Non-users reported that they would have used the InfoSpot with the platform if they had been aware of its existence. All participants reported a positive view of the digital health messages, especially animations as a health knowledge transfer tool. In conclusion, different and unintended use of the platform shows that the communities are creative in ways of utilizing the InfoSpots and gaining knowledge. The platform could have been used by more people if it had been promoted better in the communities.

## BACKGROUND

Increased health literacy gained through community health promotion activities, such as health education, may function as an accelerator towards achieving targets related to Sustainable Development Goal 3—to ensure healthy lives and to promote well-being for all at all ages—and has great potential to affect a range of other SDGs (WHO, 2017). Access to good-quality health education for disease management and prevention in the global south can be a challenge for rural people. According to the World Bank, 65% of the population in Tanzania live in rural areas (The World Bank, 2020), and healthcare workers at dispensaries or community health workers at village offices are normally the primary providers of health promotion information to such communities (MoHSW and United Republic of Tanzania, 2015). This includes information to groups or individuals, orally or via leaflets.

Tanzania is aiming for increased use of digital health and has outlined the potential of digital health education for communities to adapt healthier behaviours and increase health literacy in their Digital Health Strategy 2019–24 (MoHCDGEC, 2019). mHealth, defined as the use of mobile wireless technologies for health (WHO, 2019), can be useful to deliver health education and improve health-seeking behaviour and health-related lifestyle decisions, because they make people more reachable (Hall *et al.*, 2014). The adaptation of smartphones and tablets for health education in low-resource settings is promising (McHenry *et al.*, 2019), and health-seeking behaviour may change after a target group has been exposed to health messages that provide relevant information to the target population (Lester *et al.*, 2010; Free *et al.*, 2013). By providing relevant information, digital health can contribute to prevention and management of diseases (Ahern *et al.*, 2006).

This case study is part of the Non-Discriminating access for Digital Inclusion (DigI) project. The project provides free access to community information spots (InfoSpots) with a digital health education platform (the DigI Platform: Non-Discriminating access for Digital Inclusion, 2021), herein referred to as ‘the platform’, addressing health education gaps in rural areas of Tanzania. The platform can be viewed as a website containing animations, text and quizzes related to several diseases. All InfoSpots have one or two tablet devices for public use, and people can connect to the platform with their own devices without any costs. Lightweight webpages can also be accessed free of charge, while heavy content pages (i.e. streaming) are accessible through a voucher system. The platform is always accessible, as it is stored on a local server, and the utilization of the platform is always free.

The project was rolled out in the communities of Izazi and Migoli in 2019. The villages are located in the Iringa district of the Iringa region in Tanzania. The project has installed one main InfoSpot in each village, at the Nyerere high school in Migoli, and at the village office in Izazi. Three additional InfoSpots have been installed in the villages, in both of the local village dispensaries and in the village office in Migoli.

Within the DigI project, a quantitative study is ongoing to assess the effect of an adapted digital health education intervention, with 298 participants recruited from Migoli and Izazi. The diseases addressed in the intervention are HIV/AIDS, Tuberculosis and *Taenia solium* cysticercosis/taeniosis (TSCT). All three diseases are endemic to the Iringa region (TACAIDS and ZAC, 2017; NTLP, 2018; Ngowi *et al.*, 2019). Thus, preventive strategies are important. The intervention consists of two parts: (i) a one-time exposure to three health animated videos (animations) on a tablet device (April/May 2019) and (ii) free access to the platform in the InfoSpots, rolled out in October and November 2019 in Migoli and Izazi. The InfoSpot and the platform was promoted by project members and enumerators working with the DigI project in Migoli and Izazi prior to the rollout.

Several studies have been conducted earlier on mHealth projects in sub-Saharan Africa, primarily using text messaging (‘SMS’). L’Engle *et al.* (2013) conclude that mobile phones are suitable for health education in Tanzania, while Obasola and Mabawonku (2018) from Nigeria report that mobile phones are viewed as useful, but the internet is not useful related to maternal health education. Community health workers in Uganda have shown enthusiasm for a community-based mHealth HIV/AIDS care programme (Chang *et al.*, 2013), and healthcare workers in Tanzania report mHealth tools to be powerful and to simplify work (Shao *et al.*, 2015). A systematic review by Njoroge *et al.* (2017) from Kenya found that fewer eHealth (including mHealth) projects were implemented in marginalized areas and least urbanized counties than in urban areas. Some studies have explored animations from a health education perspective. Coetzee *et al.* (2018) conducted a study in a township in South Africa that showed that health education videos (about HIV, nutrition, alcohol and breastfeeding) provided on a tablet device are an acceptable and feasible way of health promotion. Hébert *et al.* (Hébert *et al.*, 2020) concluded that health animations, as health knowledge transfer tools, are effective for improving knowledge among health professionals in Burkina Faso. Research from Niger (Bello-Bravo *et al.*, 2019) suggests that the use of animated videos as a knowledge transfer tool is an inclusive strategy for low-literate learners, such as farmers in resource-poor settings in the global south.

To our knowledge, no studies report the use of established physical InfoSpots with free access to health education and the internet in rural sub-Saharan areas. Our case study fills this gap by exploring the participants’ perspectives and experiences related to the digital health intervention among users, non-users and key persons in Migoli and Izazi.

## METHODS

This case study aimed to explore the perspectives and experiences of rural people in Migoli and Izazi in Tanzania, with regards to being a user or a non-user of the InfoSpots with the platform. The case study is part of a mixed method study, and is described in a published protocol (Holst *et al.*, 2021).

A user would be a person that had accessed the InfoSpot and the platform at least once. A non-user would never have utilized the InfoSpot, but he or she may have been exposed to the health animations in the digital health intervention study.

A qualitative approach using content analysis was chosen (Bengtsson, 2016). We conducted 35 semi-structured interviews with people living or studying in either of the two villages to capture perspectives and experiences from users and non-users. All interviews were conducted in February 2020. The research team consisted of National Institute for Medical Research (NIMR) and University of Oslo. NIMR facilitated an interpreter and a coordinator in the field during data collection. Researchers by NIMR introduced the participants to the study in the local language. Thirty of the interviews were conducted in Swahili, two in English and three with both English and Swahili. The interviews took place in private settings at the InfoSpots and offices nearby.

### Recruitment of participants

We interviewed people from two groups:

Group 1: Participants enrolled in the quantitative study (*n* = 11). The participants were randomly sampled from participant lists from the quantitative study. All participants in this group had been exposed to the first part of the digital health intervention (the viewing of the three animations), but not all had visited the InfoSpots after rollout.

Group 2: Key people (*n* = 24) who were connected to our DigI project. This group includes InfoSpots users, i.e. villagers, healthcare workers at the dispensaries, students and teachers at the high school in Migoli, and village officers and leaders from both villages. We recruited on location, using convenient sampling (Lavrakas, 2008) around the InfoSpots in the village marketplace, school and dispensaries. As we had focal persons (village and sub-village leaders) in or around the InfoSpots related to our project, we employed snowballing to reach additional participants.

### Conduct of the interviews

During the interviews, we used a semi-structured interview guide with 11 topics (see Supplementary Annex S1). In the interviews with the non-users, we excluded the topics related to the use of InfoSpots, and rather focused on the reasons for non-use and the participants’ perspectives on digital health education, including animations. All interviews, except one, where the participant refused recording, were recorded. In addition, notes were taken during the interviews. Simultaneous translation was utilized throughout all the interviews that were conducted in Swahili in order for the non-native researcher to follow-up with probe questions. At the end of each interview, the participants were asked if they had any questions or further comments. After each interview, the research team had a debrief session where the most important topics were discussed. In this session, all unforeseen interviewee’ perspectives or experiences were also recorded in the field notes.

In Group 1, we reached data saturation point (Saunders *et al.*, 2018) after 11 interviews. In Group 2, we interviewed as many people as possible to capture the different perspectives among the participants.

### Analysis of the interviews

All interviews were thereafter transcribed and translated by a trained sociologist at NIMR with fluent language skills in both English and Swahili. The transcripts were carefully reviewed by the first author and other members of the DigI team. Thereafter, a coding frame for the data was developed, and the data were systematically coded using the software NVivo. CH derived themes of interest from the interview guide as well as new themes from the data and analysed both to contextualize the participants’ perspectives and experiences.

### Ethical aspects

This study has been assessed by the Norwegian Centre for Research Data (NSD), reference number 59643. Ethical approval from the National Institute for Medical Research (NIMR), in Tanzania, has been granted with the reference number NIMR/HQ/R.8a/Vol IX/2947.

We explained the objectives of the study to each participant and that participation was voluntary. All participants received information about the study orally, in addition to an information sheet, before they read through and signed the consent form. For the students below 18 years of age living at the boarding school, consent was provided by the teacher in addition to the participant. The participants were ensured confidentiality and anonymity and that their feedback would be handled with care and only used for research purposes.

In the quotes utilized in this paper, the participants’ age has been modified within a range of ±5, and the occupation is only stated if this does not reveal the participants identity.

## RESULTS

### Demographic information

We interviewed 35 people, 14 women and 21 men, between 15 and 55 years (see Table 1 for characteristics of the participants). Among the participants were 8 students, 5 farmers, 4 healthcare workers, 4 teachers, 4 officers/village leaders and 10 others with different occupations: fishermen, vendors, butchers, mechanics, craftsmen and community health workers. Of all the participants, 25 (71%) were classified as InfoSpot users, and 10 (29%) were non-users. All participants either went to high school, worked or lived in Izazi or Migoli. Fourteen of the participants reported to have primary education (completed or some years), 8 were in or had completed secondary education, and 13 participants reported to have post-secondary education (including 7 healthcare workers and teachers).

All participants reported that they could read and understand a leaflet with health information. The majority of the respondents reported to frequently use the internet, mostly through smartphones. WhatsApp, Facebook, Instagram, YouTube and Google were often mentioned as services and sites the participant would use.

A healthcare worker elaborated:

*I normally use internet in two main ways. First, in learning different contents in relation to my profession, disease management, and doses [of medicine]. Second, I use internet in social media as entertainment.* Healthcare worker (26 years)

Nine participants reported that they had not used internet earlier. Most were farmers and vendors, and they reported that they did not have a smartphone. All had primary education. From the users who had not used internet previously, one participant was under the age of 20, the rest was between 31 and 42 years of age.

### Non-use and use of the InfoSpots

Study participants shared their perspectives and experiences related to use and non-use of the InfoSpots and the platform. Out of five InfoSpots in Izazi and Migoli, only three were currently operative and in use. These were the main InfoSpots in Nyerere High School in Migoli and at the village office in Izazi, in addition to the InfoSpot in Izazi dispensary. At the high school and at the village office in Izazi, people accessed the platform to get health information on a daily basis.

#### InfoSpot non-users

The two InfoSpots in Migoli dispensary and Migoli village centre, both remotely connected to the high school, were not in use while the data collection for this paper was ongoing. At the dispensary, the staff had been changed since the rollout of the InfoSpot, and the knowledge on how to access the platform had not been transferred. The system was operational but not used. Two participants from the dispensary were subsequently categorized as non-users, as they had never accessed the platform earlier. In the Migoli village centre, the equipment was stored in a small shop. The radio signals could not reach this InfoSpot.

Of the 11 participants in Migoli and Izazi who were exposed to the health animations in the quantitative study, only 3 had later accessed the platform since the exposure intervention in April/May 2019. The eight others were therefore classified as non-users of the InfoSpots. We asked a participant if he had used the InfoSpot with the platform in his community, and he replied:

*No, I haven’t. Apart from the interview we did last time where you showed us the videos, we were not aware about a system installed and a platform we could access and learn things*. Villager (30 years)

Many participants, users and non-users, pointed out that people were not informed about this activity in the communities. Some participants suggested that the project needed to put more emphasis on advertisement in front of the InfoSpots and at village meetings. An officer explained:

*The contents are good with everything with it. But my concern is when introducing projects like this, you should prepare something like advertisement, a banner or flyer, so that people can easily be informed about it. I don’t know how far you reached in making something like that?* Officer (32 years)

All of the non-users reported that they did not know that the InfoSpot was open, in use and available for all. When we asked if they would have gone for health education if they had known it was open, all said yes. A fisherman explained:

*I haven’t been to visit them because I wasn’t informed, second due to the nature of what I do for a living. Most of the time I am not around at home due to economic hardships. Regardless of that, if I had been aware of the platform, I would have visited it.* Fisherman (28 years)

#### InfoSpot users

The 25 participants classified as InfoSpot users reported variations in use patterns. Some of the users, the leaders and teachers, had been using the InfoSpots to access a variety of webpages on the internet, in addition to the platform with the health messages, but 21 of the users reported only using the platform with the health education. The onset of use among the users was gradual. Four of the users had started to use the InfoSpot when it was installed in August 2019, by only accessing the internet and not the platform. Eight of the users started when the platform was rolled out later in November, and 13 users reported using the InfoSpot for the first time in December, January or early February. Related to the frequency of the use, 13 users said that they used the InfoSpot daily or weekly, all of whom had secondary school or higher education, while 12 users reported to use the InfoSpot occasionally. The difference in use is elaborated below.

#### Nyerere High School

At Nyerere High School, a secondary boarding school with two levels (O' Level with Form 1–4 and A' Level with Forms 5 and 6) and more than 1000 students and 25–30 teachers, the system was working well, with all features intact. Two tablets were in use in the school’s IT classroom. The students were not allowed to have their own smartphones at this boarding school. Some teachers reported to have accessed the internet and platform with their own devices. The students reported that they only used the platform, while some teachers reported that they had also accessed other websites on the internet. The teachers reported using different components of the platform in class: They would use the written health messages to prepare for class and use the quiz for themselves and in class for the students. A teacher elaborated on their use of the InfoSpot and platform:

*Yes, I use it when I’m at work; but sometimes, after working hours, we usually come back here for remedial classes or to supervise students, so I usually decide to read a topic, for example, on tuberculosis; and to measure if I read well, I take a quiz, submit it and get feedback, so it helps.* Teacher (27 years)

The students reported using the platform to learn about the diseases by viewing the animations, taking the quiz and reading the key messages. We learnt that students also used both English and Swahili when they accessed the health content on the platform:

*I watched using both languages. For example, if I’m watching it in English, and let’s say I don’t understand causes of disease written in English, I just translate it to Swahili. That’s what I do, so I use both languages.* Student (15 years)

The majority of the students interviewed (six out of eight) reported using both languages while using the platform. Another student explained:

*It is easy because there are two languages on the platform, Swahili and English. So, anyone who has a problem with English can use Swahili and understand it well, and one with problems with Swahili can use English.* Student (16 years)

All students who reported using a smartphone back home told us that the platform was simple to use. A student explained:

*It is not hard [to use the platform] because the majority of our generation have knowledge of the internet, so it’s easier to visit a platform like this without any complications, and search for things in a user-friendly manner.* Student (20 years)

A teacher elaborated on the user-friendliness according to age:

*Yeah! It’s very easy, but I found some difficulties in some of the students who are still young, for example form one and form two. Most of them are not familiar but for the form three and the form four students, they are familiar.* Teacher (27 years)

#### Izazi village office

In the Izazi village office, the system was operational, and it was used both when the DigI project topped up the internet credits allowing full internet access to be available and when users were only given access to the platform. Two tablets were in use. In this InfoSpot, the use was different than at the high school. The leaders and users with more formal education reported using all features of the InfoSpot; they were going online to use the internet and WhatsApp, in addition to accessing the platform for health education for themselves and for teaching others. Both the available tablets and users’ own smartphones were used in this InfoSpot. Different groups, including farmers, officers and community health workers (not health professionals, but volunteers in the community), used the InfoSpot. A community health worker pointed out that the use of the InfoSpot had reduced their health education workload:

*Well, for us it has reduced our burden to teach people; instead of us teaching them about health by using words and talking, now they can just watch informative videos for diseases like Tegu [Swahili for pork tapeworm/cysticercosis], HIV/AIDS, and TB, and I think it’s easy for them to learn and understand this way.* Community health worker (22 years)

The same participant explained that she used the platform to rehearse before teaching others:

*Since December I used to come frequently! Because most of the time, I have classes to teach, so I come here to learn more before I teach in class.* Community health worker (22 years)

We met a farmer who came into the village office to meet the village leader. After a talk in the office, the farmer was given the tablet device with the animations ready to watch. Most of the participants in the Izazi village office reported that the InfoSpots were easy to use, while some of the participants reported that a leader, in order to access the platform and to see the animations, guided them. The farmer explained:

*Well, to be honest, I am not competent with electronic gadgets, so there was a person who was helping me to use it.* Farmer (42 years)

Several participants reported on the same topic:

*There were ladies guiding us, they showed us how to navigate from one video to another.* Villager (25 years)

Another villager explained it this way when we asked if the tablets were easy to use for the people in the community:

*Um, it depends on how familiar the person is. For the knowledgeable ones, it becomes easier and vice versa, that is true.* Villager (27 years)

#### Izazi dispensary

At Izazi dispensary, the platform was available and accessible, but the tablet used in the very beginning after the rollout of the platform was no longer in use by the patients in the waiting area, due to risk of contamination between patients. The tablet was now rather used by the healthcare workers to refresh their own memory and to educate groups of people in the community. We learnt from the healthcare workers that they would bring the tablets with them when going on outreach and show animations for people out in the rural areas. The animations were stored as offline content on the tablet. In addition to that particular use, the healthcare workers would place the tablet on a small table in the waiting area for the health education of waiting patients. A healthcare worker explained how they would use the tablets to save time, and the animations for public health education, and when encouraging people to change their health behaviour:

*For example, before the tablets, we were having the program that provides education each morning, and from there, we go to provide services. You may find that we talked about cholera one day and about another disease another day. We were doing… like, public health education. But after receiving the tablets, we put them [patients] in groups of five people and we showed them clips which are in series. And sometimes, when we see a person coughing without covering the mouth, we show him the clip and they understand.* Healthcare worker (32 years)

A healthcare worker explained that they would use the platform to refresh their own memory:

*It helps us a lot because sometimes it reminds even us of diseases that we learned about in school a while ago.* Healthcare worker (41 years)

A third healthcare worker pointed out the low levels of digital skills in the rural areas, and explored reasons for non-use:

*People in the communities need training in how to use both the device and platform. Some have never used it before, not even smartphones.* Healthcare worker (26 years)

### Perspectives on animations as a learning method

Almost all participants (33 out of 35), whether they were InfoSpot users or not, had viewed the animations at least once and reported a positive view of digital health education and the use of animations for health knowledge transfer. Many of the participants pointed out that it was easy to learn from the animations, as the viewer could see the actions of the characters in the animations, and that this had an impact on the learning. A vendor in Migoli explained:

*You see, watching a video, you see each and everything, all demonstrations. For instance, a video says, ‘in order to prevent yourself from this disease, you have to do this and that by seeing the actions’, but when you are just reading a leaflet, it’s common to forget details. So, the best way to learn is by TV. Even myself, after watching the videos, I grasped new things.* Villager (19 years)

A student said it this way:

*This approach in learning is very nice because it creates a long term memory, different from reading only.* Student (19 years)

### Sharing health messages and recommending others to use the platform

The vast majority of participants reported that they had shared their new knowledge obtained through watching the animations with others, often friends or family. All InfoSpot users had recommended using the platform to others. A farmer explained why he had recommended to others to use the platform:

*I thought it was the best method because, directly, the service is at the hospital where we can get tested. The demonstrations in the video speak a lot, that’s why I encouraged others to come watch, learn, and understand.* Farmer (32 years)

A student explained how he/she had tried to recruit fellow student to access the InfoSpot:

*Yeah! I am trying to convince my fellow student to use this, but I succeeded to get only ten students who frequently visit [the platform] due to insufficient number of tablets.* Student (21 years)

A teacher explained how he had downloaded the content on his smartphone to show others in the community:

*I used to download videos and save them and watch them offline and share with the family members.* Teacher (32 years)

## DISCUSSION

The digital health intervention in the overarching DigI project is a unique combination of the provision of health education in animation format, access to the internet and a digital health education platform. Smartphones, laptops and desktop computers to access the internet are more commonly used among the high school students (although not at school) and the participants with more education (healthcare workers, teachers, officers) than for the less formally educated participants (farmers, fishermen, craftsmen and vendors, among others). All of the participants who reported never having used the internet before had primary school education only. This is part of what is commonly known as the digital divide referring to the gap between those who are able to benefit from the digital age compared to those who are not (Hilbert, 2011). In the literature, we find technological, immaterial, material, social and educational types of inequalities that may account for the digital divide (Van Dijk, 2006). All these types of inequality can be used to explain the difference in access to technology and the internet across regions, and are highly relevant for this case study, related to the use of the InfoSpots and perspectives regarding the digital health intervention. In Obasola and Mabawonku’s study, the perceptions and willingness to use ICT for maternal and child health information was explored, and almost 70% of the mothers reported the internet not to be useful, especially in the rural areas where connectivity, and use of internet, was low. In the DigI project, we deliberately addressed areas where internet connectivity and usage were low. It became clear that besides the lack of connectivity, other deficiencies limited the farmers’ technological opportunities, as the majority of non-users had limited or no access to a smartphone or other devices to access digital information.

When the platform in this project was rolled out in the InfoSpots, a small launch party in both Migoli and Izazi celebrated the opening. The interviews were undertaken in February 2020, leaving only a short time for adaptation of the utilization of the platform in the villages. The time between November (2019) and February (2020) was characterized by more rainfall than normal (FAO, 2020), which could have contributed to a higher workload for the farmers. Being a farmer in rural Tanzania is demanding, and the need for internet on a daily basis may be limited. However, and unlike in the Nigerian study (Obasola and Mabawonku, 2018), our non-users indicated that they would have used the InfoSpots for health education if they had known that they were available, and therefore, we assume that they perceive the platform to be useful.

As a recommendation, the InfoSpots could have been promoted better in the villages, for example at village meetings and by posters in and around the InfoSpots. At the same time, it is hard to reach all village people, as the different seasons demand different efforts in animal husbandry and harvesting. The DigI project did not directly target farmers or vendors, but we found that they were eager to learn and would recommend the use of the InfoSpots with the health animations to others. The same findings have been reported in a study from Niger, where farmers reported higher preferences for animations as a learning method and would recommend animations as a form of teaching for others (Bello-Bravo *et al.*, 2019).

### Different use of the InfoSpots across groups in the communities

Our case study found that utilization of the InfoSpots differed by location, age and occupation. Reports by students on the non-intentional use of animations in both languages, e.g. to practice English, was surprising and unexpected. The applicability for other learning scenarios shows that providing a digital health intervention addresses multiple outcomes. In this case, learning the English language from a digital platform can be seen as a great add-on, which can help facilitate the aim of increasing health knowledge, general education and digital literacy. It also indicates the large potential for the younger generation to take advantage of such community InfoSpots. All participants from the high school area reported that tablets were easy to use, and that they navigated within the platform themselves, which suggests that such health interventions can be especially beneficial to secondary and high schools.

In the Izazi village office InfoSpot, the leaders and community health workers often introduced the animations to the villagers who came into the offices for various reasons and guided interactions with the platform. Thus, the digital health content was provided in a supervised form, which may limit opportunities for individuals to increase their digital literacy skills, by playing around with the tablet to explore the platform. Supervision may occur out of necessity and again reflects the digital divide and the gap in digital and technological literacy. However, the guidance of villagers into the platform shows creativity and enthusiasm from the leaders in the community for the ways to adapt and facilitate community education. It could very well be that this represents the first step towards an increase in both health knowledge and digital skills.

Patients at the dispensary were not allowed to use the tablet while waiting for their appointment due to the risk of contamination. Our team subsequently followed the local recommendation and placed TV screens with animations for health education in health facilities as a more appropriate solution than tablets. Our findings that healthcare workers and community health workers found that the use of tablets reduced their workload by saving time on health promoting activities, supports previous findings from South Africa, where video teaching tools helped community health workers with administrative duties and reduced the time of household visits (Coetzee *et al.*, 2018). The community health workers could do the needed administration while the households they visited were busy seeing the health video. In our case, the healthcare workers would show the animations to groups while doing other work in the dispensary. Healthcare workers interviewed in this case study also reported using the animations to refresh their own knowledge, especially related to diseases that do not have a public focus, i.e. TSCT. Refreshing knowledge as unintended use shows that the value of the platform was also beneficial to healthcare workers and indicates yet another creative use of the platform in rural communities. Knowledge updates for community health workers and professional healthcare workers, as well as digital health information on new or emerging diseases, for example COVID-19, may strengthen the healthcare workers’ knowledge. Thus, our case study supports that providing platforms in communities may represent an important part of community health education.

### Limitations

This case study has several limitations. The answers that we received may have been biased. As the interviewer represented the donor organization, people’s answers could have been influenced by that. In rural TZ, people may ‘agree’ with you, saying yes because they think that it is what they should say. For example, some of the participants answered ‘yes’ to the question, ‘Have you visited any of the InfoSpots around the village?’ But when we asked which one and when, we could not get an answer. It may very well be that the participant wanted to show interest and tried not to be rude. We utilized probing questions to deepen the knowledge on the use and non-use and to explore the participants’ personal thoughts and perceptions about the project.

At the school, we had to ask if we could speak to some of the girls after having interviewed five boys in a row. This may raise a question about the sampling. We were introduced to the students by the teacher. It may have been important for the teachers to give us great examples of the students, since the DigI team had already provided equipment and access to the internet.

## CONCLUSION

The findings from this case study show a variation in the use of the platform through InfoSpots among the various user groups. What is in common is the perceived value of the health education through animations, which was seen as easy to understand. Young participants, like the high school students, found the InfoSpots easy to use and had no difficulties navigating the platform with the available tablet devices. This group used the platform for health education and for learning English, while their teachers used the platform to prepare for class and to surf the internet. More formally educated participants, village leaders or healthcare workers often guided less educated participants within the platform. The use of InfoSpots in Izazi depends on the more educated users digital skills, while the InfoSpot in Migoli is more independently used, indicating that the younger people and more educated groups benefit more from the platform than the older and less educated participants, from a digital skills perspective. Healthcare workers in the dispensary used the platform to refresh their own memory, in addition to educating groups in the communities and village meetings. Community health workers further reported that the animations contributed to saving time on health promotion activities. These findings demonstrate that the communities are creative in the ways of using the InfoSpot and the platform for educational gains, and thus achieve both health knowledge, other education and digital skills. The non-users interviewed in this case study pointed out that they would have used the InfoSpots if they had known they existed, which points to the value and importance of promoting digital health interventions.

All participants in this case study had a positive perception of the digital health education intervention. The use of animations to transfer health messages to individuals and communities was especially appreciated. In conclusion, this case study demonstrates that animations for health knowledge transfer through InfoSpots in the communities are appropriate for providing health education. Digital health promotion through animations has the potential to reach both more and less educated groups in rural Tanzania.

## SUPPLEMENTARY MATERIAL

Supplementary material is available at *Health Promotion International* online.

## Supplementary Material

daab187_Supplementary_Data
