# Supplementary material for: Utilizing community InfoSpots for health education: perspectives and experiences in Migoli and Izazi, Tanzania
Source: Health Promot Int. 2021 Dec 13;38(4):daab187. doi: 10.1093/heapro/daab187 (PMC10439510; doi:10.1093/heapro/daab187)
Supplement: daab187_Supplementary_Data [file daab187_supplementary_data.docx]

# Annex 1 - Interview guide semi-structured interviews (ENG)

1. Do you know how to read a health information leaflet? YES/ NO

2. Have you used the InfoSpot in the village?

YES / NO

- if not – why not, please explain? If yes, please tell me about your experiences with it.

3. If yes in no 2: Have you looked at any of the free digital health messages?

YES/NO - (if yes, which ones?)

4. If yes in no 2: When did you use the InfoSpots, first and last time?

5. If yes in no 2: How often have you used it?

6. What do you think of the digital health message(s)?

Please explain - at least one negative thing and one positive thing.

7. What did you learn from them? (please tell me what you remember the most)

8. Have you explained the messages to any of your family members? (If yes, please tell me about your experiences with it.) (if not, why not?)

9. What can we improve? Please tell us what you think is not functioning optimally.

10. What is not user friendly? Please tell me if there are anything that you find difficult when using the InfoSpots.

11. Would you recommend it for someone else to use? Why? Why not?
